# *Sirenomelia* Phenotype in *Bmp7;Shh* Compound Mutants: A Novel Experimental Model for Studies of Caudal Body Malformations

**DOI:** 10.1371/journal.pone.0044962

**Published:** 2012-09-17

**Authors:** Carlos Garrido-Allepuz, Domingo González-Lamuño, Maria A. Ros

**Affiliations:** 1 Instituto de Biomedicina y Biotecnología de Cantabria (IBBTEC), CSIC-SODERCAN-Universidad de Cantabria, Santander, Spain; 2 Instituto de Formación e Investigación Marqués de Valdecilla (IFIMAV) and División de Pediatría, Hospital Marqués de Valdecilla-Universidad de Cantabria, Santander, Spain; Ecole Normale Supérieure de Lyon, France

## Abstract

*Sirenomelia* is a severe congenital malformation of the lower body characterized by the fusion of the legs into a single lower limb. This striking external phenotype consistently associates severe visceral abnormalities, most commonly of the kidneys, intestine, and genitalia that generally make the condition lethal. Although the causes of *sirenomelia* remain unknown, clinical studies have yielded two major hypotheses: i) a primary defect in the generation of caudal mesoderm, ii) a primary vascular defect that leaves the caudal part of the embryo hypoperfused. Interestingly, *Sirenomelia* has been shown to have a genetic basis in mice, and although it has been considered a sporadic condition in humans, recently some possible familial cases have been reported. Here, we report that the removal of one or both functional alleles of *Shh* from the *Bmp7*-null background leads to a *sirenomelia* phenotype that faithfully replicates the constellation of external and internal malformations, typical of the human condition. These mutants represent an invaluable model in which we have analyzed the pathogenesis of *sirenomelia*. We show that the signaling defect predominantly impacts the morphogenesis of the hindgut and the development of the caudal end of the dorsal aortas. The deficient formation of ventral midline structures, including the interlimb mesoderm caudal to the umbilicus, leads to the approximation and merging of the hindlimb fields. Our study provides new insights for the understanding of the mechanisms resulting in caudal body malformations, including *sirenomelia*.

## Introduction

*Sirenomelia*, or *sirenomelia* sequence, is a rare (1.1–4.2 births of 100.000) and striking congenital malformation of the lower body characterized by the fusion of the lower limbs [1], [2], [3], [4], [5], [6] . This feature, which is pathognomonic for the diagnosis, gives fetuses and newborns a mermaid appearance, thence the alternative name of *Mermaid Syndrome*. *Sirenomelia* is a multisystemic condition because of the constant association of significant anomalies, mostly but not exclusively of the caudal body. It is a severe condition, usually lethal in the perinatal period [3], [4] although less severe cases have recently survived with appropriate reconstructive surgery [7].

*Sirenomelia* shows an ample spectrum of clinical presentations due to the variability in the degree of fusion of the lower limbs and in the combination of visceral malformations [2], [4], [8], [9], [10]. Without exception, genitourinary and gastrointestinal malformations are found in *sirenomelia*, since they are features secondary to oligohydramnios [5], [8], [11], [12]. Agenesis of the terminal colon with imperforate anus occurs in the majority of cases [3], [4], [5], [8], [11]. The kidneys, ureters, urinary bladder and external genitalia are always hypoplastic in variable degree whereas the gonads are usually unaltered. Characteristically, the umbilical cord has a single artery and major anomalies of the abdominal arterial system are also common [3], [4], [10], [13].

Although the causes of human *sirenomelia* remain unknown, clinical studies have yield two main pathogenetic hypotheses: the defective blastogenesis hypothesis and the vascular steal hypothesis. The defective blastogenesis hypothesis is supported by the overall malformation of the caudal body in *sirenomelia*. This suggests an early defect, probably a deficient generation of mesodermal precursors at late gastrula stages, which globally impairs the development of the caudal embryonic body [14], [15], [16]. Under this view, *sirenomelia* is considered a severe manifestation of the complex malformation called Caudal Dysgenesis (CD; OMIM 600145; formerly referred to as Caudal Regression Syndrome). CD describes a heterogeneous variable association of malformations of the lower body that always includes some degree of sacral agenesis [12], [15], [17], [18], [19], [20].

The vascular steal hypothesis posits a vascular origin for *sirenomelia*. This is based on the observation that fetuses with *sirenomelia* almost invariably exhibit a single umbilical artery (SUA), instead of the normal two [13], [21]. The SUA originates quite high from the abdominal aorta, usually immediately beneath the celiac branch, and below its origin the abdominal aorta is highly hypoplastic and lacks most of its branches. It has been proposed that the SUA diverts the normal blood flow to the placenta leaving the lower part of the body with a severely deficient circulation incompatible with normal development [2], [8], [21]. The SUA has also been referred to as persistent vitelline artery to indicate its possible origin in the vitelline plexus and its presence is considered by some authors as pathognomonic of *sirenomelia* although it has occasionally been reported in other conditions [2], [8], [14], [18], [21], [22], [23], [24]. In this view, *sirenomelia* and CD are considered two separate conditions. Therefore, some debate remains in the clinic on whether *sirenomelia* and CD are separate conditions or just different degrees of the same multisystemic malformation [12], [24].

Besides clinical data, experimental data in mouse indicate a genetic origin for *sirenomelia* as this malformation has been found in several genetically modified mouse strains with either loss-of-function of bone morphogenetic protein (Bmp) signaling or with gain-of-function of retinoic acid (RA) signaling [25], [26]. Yet, human *sirenomelia* has mainly been considered sporadic since familial cases have only been reported very recently [27]. However, it should be noted that the lack of evidence for a major genetic influence in human mermaid syndrome may rely, at least in part, on the poor documentation of abortions [4], [28].

The implication of Bmp signaling in *sirenomelia* came from the observation that the double *Bmp7;Tsg* (*Twisted gastrulation*) mutant mouse displayed this malformation [26]. Tsg is a modulator of Bmp signaling that in the caudal body positively influences Bmp signaling. Therefore, the removal of one or both copies of *Tsg* from the *Bmp7* background further reduces Bmp signaling resulting in caudal abnormalities and *sirenomelia* [26].

The implication of RA signaling in *sirenomelia* came from the observation that excess of administration of RA to pregnant mice led to caudal malformation of the offspring including *sirenomelia* in some cases [29]. This has been confirmed by the occasional presentation of *sirenomelia* in the genetic disruption of *Cyp26a1*, an enzyme that degrades RA [25], [30] and in the disruption of other genes that interact with the Cyp26 family of enzymes [31], [32], [33], [34].

Unfortunately, the low penetrance and early embryonic lethality of *Bmp7;Tsg* and of *Cyp26a1* mutants exclude them as suitable models of human *sirenomelia*. The availability of an animal model of *sirenomelia* would be an invaluable tool to unravel its pathogenesis and it could provide insights on the development of the caudal body. Such a model will also permit to analyze the relationship between *sirenomelia* and CD and the influence of epigenetic factors including RA and maternal diabetes.

Here we identify and characterize the *sirenomelia* phenotype in *Bmp7;Shh* compound mutants. We show that *Bmp7^−/−^;Shh^−/−^* and *Bmp7^−/−^;Shh^+/−^* mutants conjointly replicate the constellation of external and internal malformations in the human condition and thus we propose these mutants as the most suitable animal model for human *sirenomelia* to date.

The analysis we have performed shows the early onset of the malformation that is detectable at E9.5. The atrophic hindgut and cloaca together with the defective development of the caudal end of the dorsal aortas, which form by vasculogenesis [35], [36], result in a defective midline and ventral mesoderm caudal to the umbilicus that leads to the junction of the hindlimb fields. Our results suggest that the lower limb fusion in *sirenomelia*, although giving the name to the condition, is a secondary event and that *sirenomelia* phenotypes may occur without significant impact in dorsal structures.

## Materials and Methods

### Mutant mice

Studies with mice were performed strictly following the EU regulations and 3R principles. The *Bmp7* [University of Oxford, Oxford, UK; 37] and *Shh* [University Hospital Basel, Department of Biomedicine, Switzerland; 38] mutant mice lines used in this study were kindly provided by Elisabeth Robertson (University of Oxford, Oxford, UK) and Rolf Zeller (University of Basel, Switzerland) respectively. The mice were maintained on a mixed genetic background and genotyped using tail or embryonic membranes biopsies as previously reported. Wild type and mutant embryos of the desired embryonic stage were obtained following standard protocols.

### Skeletal preparations, X-Gal staining and *in situ* hybridization

After removing skin and viscera, mouse embryos were fixed in 95% ethanol. Alizarin Red and Alcian blue skeletal staining was performed according to standard protocols cleared by KOH treatment and stored in glycerol. X-Gal staining in Whole-mount embryos was performed as previously described [37]. After staining, the embryos were fixed and processed for inclusion in paraffin if desired.

In situ hybridization (ISH) using digoxigenin labeled antisense riboprobes was performed in whole-mount and in sections [39]. The probes for mouse mRNA used were *Shh*, *Bmp4*, *Pitx1*, *Islet1*, *Fgf8*, *Tbx4*, *Cdh5*, *Lmx1b*, *Msx2*, [kindly provided by G. Martin (University of California at San Francisco, San Francisco, USA), B. Robert (Institut Pasteur, Paris, France), P. Bovolenta (Instituto Cajal, CSIC, Madrid, Spain), M. Logan (National Institute for Medical Research, London, England), and C. Tabin (Harvard Medical School, Boston, USA)].

### Semithin sections

E8.5 and E9.5 embryos were fixed in Glutaraldehyde, embedded in araldite, sectioned (1 micron thick) and stained with Methylene Blue following standard protocols.

### Cell death and cell proliferation assays

Detection of cell death in sections of paraffin-embedded tissue was performed using terminal deoxynucleotidyl transferase mediated dUTP nick-end labelling (TUNEL) with the Apoptag Fluorescein Direct In Situ Apoptosis Detection Kit (Intergen) and following the manufactured instructions. Detection of cell proliferation in sections was performed by immunohistochemical assay using the anti phosphorylated histone H3 antibody (rabbit polyclonal Phospho H3 from Upstate Biotechnology, USA) diluted at 1/100.

## Results

### Reduction of *Shh* from the *Bmp7* null background results in *sirenomelia*

In the context of other studies on limb development [40] we found that the reduction of one or both *Shh* functional alleles from the *Bmp7*-null background led to *sirenomelia*. To study this phenotype in depth, mice heterozygous for the *Bmp7* null allele (*Bmp7^+/−^*) were crossed to mice heterozygous for the *Shh* null allele (*Shh^+/−^*) and the double heterozygous (*Bmp7^+/−^*; *Shh^+/−^*) mice obtained were subsequently crossed between them.

From these crosses, the *sirenomelia* phenotype was found restricted to embryos and neonates with the *Bmp7^−/−^;Shh^−/−^* and *Bmp7^−/−^;Shh^+/−^* genotypes. Single *Bmp7^−/−^* and single *Shh^−/−^* mutants displayed their previously described phenotypes [38], [41], [42] whereas single heterozygous and double heterozygous were phenotypically normal. Interestingly, *Bmp7^+/−^;Shh^−/−^* displayed a phenotype indistinguishable from that of *Shh^−/−^* mutants that do not display *sirenomelia* (not shown) indicating that the dose of *Bmp7* is the limiting factor for the this malformation.

Up to E12.5 all genotypes were recovered at the expected Mendelian ratios, but subsequently the frequency of recovery of double homozygous mutants (*Bmp7^−/−^;Shh^−/−^*) progressively declined to be less than 2% at late gestational stages (6.25% expected). Thus, double homozygous shows an earlier embryonic lethality than single *Bmp7^−/−^* mutants, which are recovered at the expected Mendelian rates at birth and to single *Shh^−/−^* mutants, which die at the end of the gestational period. Of the 170 neonates obtained, only 3 were double homozygous mutants while *Bmp7^−/−^;Shh^+/−^* were recovered at the expected Mendelian ratio (Table S1). These two types of mutants died in the first few hours after birth due to the multisystemic visceral malformations.

**Figure 1:**
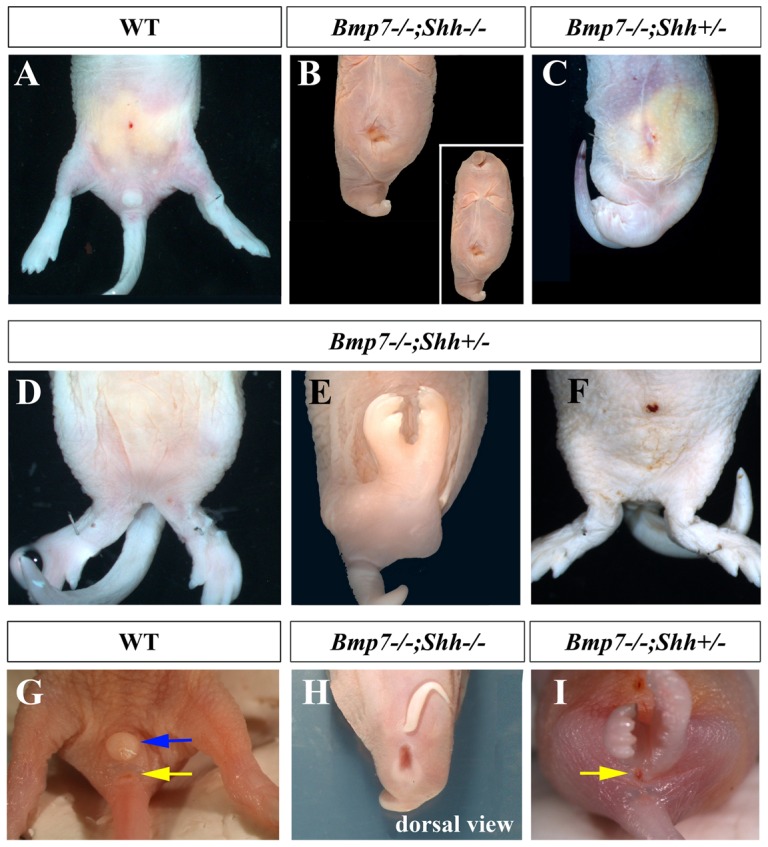
*Sirenomelia* in *Bmp7^−/−^;Shh^−/−^* and *Bmp7^−/−^;Shh^+/−^* compound mutants. (A) Ventral view of the caudal body of a wild type newborn. (B) Severe *sirenomelia* typical of *Bmp7^−/−^;Shh^−/−^* mutants. Note the centrally positioned atrophic hindlimb and also the typical features of *Shh* mutants including proboscide, cyclopia and truncated forelimbs (insert in B). (C) Milder *sirenomelia* typical of *Bmp7^−/−^;Shh^+/−^* mutants in which the two hindlimbs can still be distinguished. (D and E) *Bmp7^−/−^;Shh^+/−^* neonates showing different degrees of fusion of the hindlimbs. (F) *Bmp7^−/−^;Shh^+/−^* neonate that does not display a clear *sirenomelia* but showing midline approximation of the hindlimbs. (G) Normal appearance of the genital tubercle and anus in a wild type neonate. (H) Dorsal view of a double homozygous mutant showing imperforate anus. (I) Imperforate anus and absence of genital tubercle in a *Bmp7^−/−^;Shh^+/−^* newborn. Note the presence of a practically normal tail in *Bmp7^−/−^;Shh^+/−^* newborns. Genotypes indicated at the top of the panels. Blue arrow points to the genital tubercle and the yellow arrow to the anus.

Double null *Bmp7^−/−^;Shh^−/−^* mutants displayed *sirenomelia* with complete penetrance (100%) in addition to the typical traits of the *Shh* null phenotype such as cyclopia, proboscide and truncated forelimbs Fig. 1B; [38]. *Bmp7^−/−^;Shh^+/−^* mutants, in which one functional copy of *Shh* remains, displayed a milder form of *sirenomelia* with about 50% penetrance (11 out of 21 neonates; Fig. 1C). The hindlimb fusion in *Bmp7^−/−^;Shh^+/−^* mutants ranged from a partial merging at proximal/tight level (Fig. 1D) to a complete fusion all along the proximo-distal length of the hindlimbs (Fig. 1E). It should be mentioned that in those *Bmp7^−/−^;Shh^+/−^* mutants that did not display a clear mermaid phenotype, the position of the hindlimbs was closer to the ventral midline than normal (compare panel A with F in Fig. 1).

External inspection of double homozygous mutants neonates, in comparison with wild type littermates, revealed a complete absence of genital tubercle and imperforate cloacal membrane (compare Fig. 1G with Fig. 1H). *Bmp7^−/−^;Shh^+/−^* mutants displayed a variable range of underdeveloped genital tubercle and anal malformations including imperforated and prolapsed anus (Fig. 1I). The rudimentary genital tubercle and any evidence of anus, if present, were always located caudal to the fused hindlimbs (Fig. 1H–I).

### Skeletal phenotype of *sirenomelia* mutants

**Figure 2:**
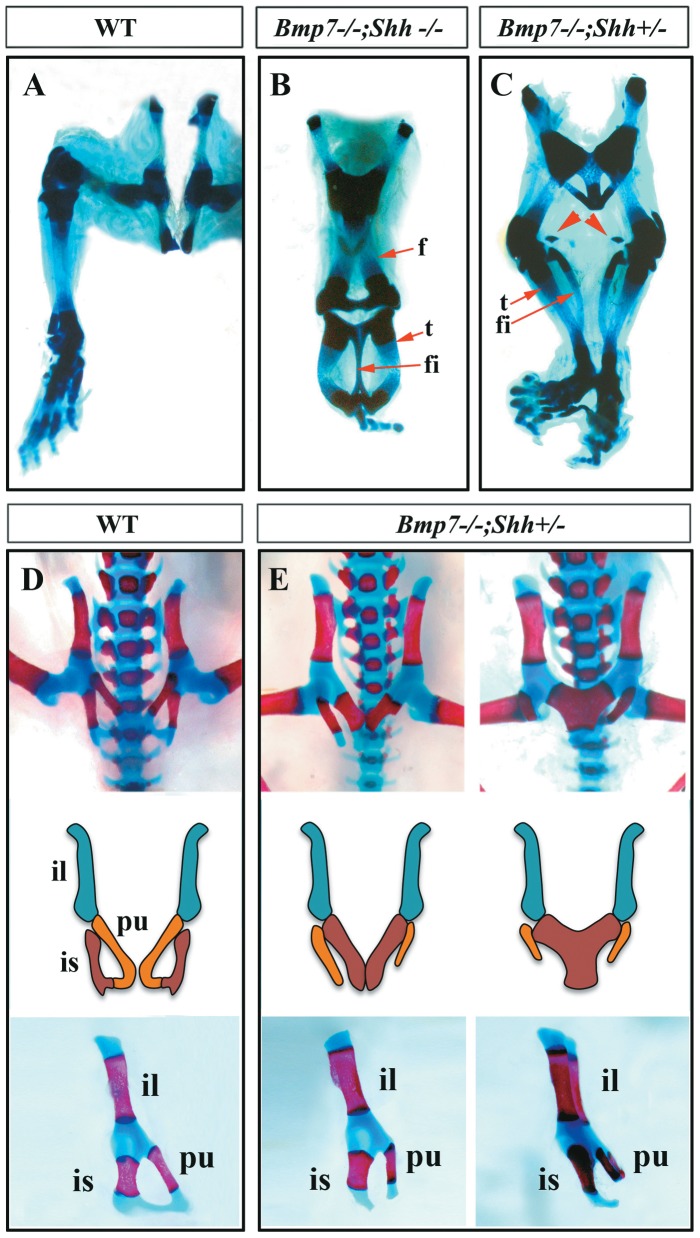
Skeletal analysis of *sirenomelia* phenotypes at birth. (A–C) Ventral views of Victoria Blue cartilage staining of representative wild type (A), *Bmp7^−/−^;Shh^−/−^* homozygous (B) and *Bmp7^−/−^;Shh^+/−^* mutant (C). Note the isquia, proximal femora (arrow) and fibula (f) fusion in double homozygous (B) and the medial position of the fibulae due to the absence of hindlimb rotation (B–C). (D and E) Ventral view, with corresponding scheme below, and lateral view of Alcian Blue and Alizarin Red stained pelvis of wild type (D) and *Bmp7^−/−^;Shh^+/−^* neonates (E). The abnormal morphology of the hip is characterized by medial approximation and fusion of the ischia, truncation of the pubes but normal sacrum (E). Abbreviations: f: femur; fi: fibula; il: illium; is: ischium; pu: pubis; t: tibia. The arrowheads in C point to the patellae.

To examine the skeletal morphology of the fused hindlimbs, we performed skeletal preparations of neonates (Alizarin red and Alcian blue and Victoria blue staining, see M&M). The skeletal preparations, in comparison with wild type (Fig. 2A), demonstrated that the single medial hindlimb typical of the severe *sirenomelia* of double homozygous (*Bmp7^−/−^;Shh^−/−^*; Fig. 2B) was the result of the fusion of two independent hindlimbs. It consisted of two proximally fused femora followed by two tibia arranged in parallel with a single fibula between them. At its proximal (knee) and distal (ankle) ends it was possible to discern that the single fibula derived from the fusion of the two bilateral fibulae. In the foot one or two digits were scored as corresponded to the *Shh* mutant phenotype that only displays one digit in each hindlimb [43], [44]. Interestingly, the zeugopod (intermediate limb segment) showed an improved morphology compared to *Shh* null embryos [43], [44] indicating that, at least part of the *Shh*-limb phenotype was due to increased Bmp signaling (Fig. S1). The hindlimb fusion exhibited by *Bmp7^−/−^;Shh^−/−^* double homozygous corresponds to Types IV–V in Stocker and Heifetz classification [3], [10].

In *Bmp7^−/−^;Shh^+/−^* mutants the hindlimb fusion was mainly of soft tissue with each of the fused hindlimbs showing a complete set of skeletal elements (Fig. 2C). In the majority of cases the two fused limbs diverged from the ankle and nine or ten digits were scored depending on whether the two pinky digits, which were in medial position, were fused. Bone fusion between tarsal-metatarsal elements of the two bilateral feet was also observed (Fig. 2C). The *sirenomelia* of *Bmp7^−/−^;Shh^+/−^* mutants can be classified as Type I of Stocker and Heifetz classification [3], [10].

Importantly, all degrees of *sirenomelia* showed a characteristic absence of hindlimb rotation that resulted in the medial position of the fibulae (or single fibula) between the two tibiae (Fig. 2B–C). The patella, when present, was consistently found in a luxated medial position (arrowheads in Fig. 2C) and the soles of the feet faced anteriorly. This very same skeletal arrangement is found in human cases of *sirenomelia* [4], [10], [28]. An early merging of the left and right hindlimb fields in the midline along their posterior borders would prevent the subsequent rotation of the hindlimbs.

Abnormalities of the pelvic skeleton were also scored in mermaid newborns (Fig. 2D–E). The pelvic (hip) bone is formed by the fusion of three distinctly developed skeletal elements: the ilium, the ischium and the pubis (see drawing in Fig. 2D). *Bmp7^−/−^;Shh^−/−^* double homozygous showed the complete absence of caudal vertebral column described for the *Shh* mutant phenotype ([38], data not shown) whereas the vertebral column, the sacrum and the tail showed a normal development in *Bmp7^−/−^;Shh^+/−^* mutants except for the distal kinked tail typical of *Bmp7* homozygous mutants (data not shown). Interestingly, *Bmp7^−/−^;Shh^+/−^* mutants showed pelvic defects characterized by a variable degree of truncation of the pubis and midline fusion of the two bilateral ischia whereas the ilia were normal (Fig. 2E). This led to an anomalous lesser pelvis that must impact the perineum and hindlimb musculature. This particular morphology of the pelvis, with both ischia fused in the midline and the pubes laterally separated, likely derives from the impossibility of the fused hindlimbs to rotate.

To sum up, our analysis shows a variable degree of approximation and fusion of the leg and pelvic skeletal elements in correlation with the genotype; fusion of leg bones was only observed in double homozygous. In all cases there is a constant absence of the normal rotation of the hindlimbs, which influences the arrangement of the skeletal elements including the pelvic bone. Finally, it should be stressed that, the mild *sirenomelia* of *Bmp7^−/−^;Shh^+/−^* occurs with a normal development of the sacrum and tail.

### Visceral phenotype of *sirenomelia* mutants

**Figure 3:**
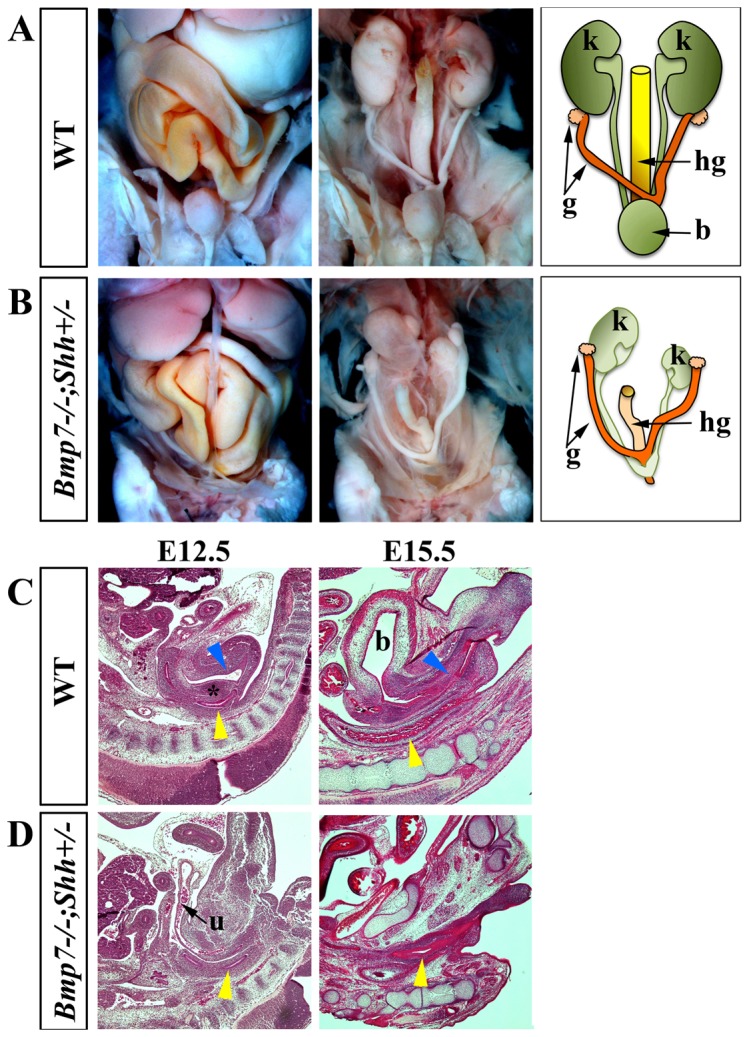
Visceral analysis in wild type and mutants. (A–B) Autopsy preparations of abdominal viscera in newborns. Superficial (left panel), profound (medium panel) and corresponding scheme (right panel) are shown. Mutant embryos exhibit relatively normal stomach and midgut but hypoplastic kidneys and hindgut and absence of bladder. (C–D) H&E-stained mid sagittal sections of wild-type (C) and *Bmp7^−/−^;Shh^+/−^* mutants (D) at the embryonic stage indicated at the top. The yellow arrows indicate the hindgut and the blue arrows the urethra. Note the absence of urogenital duct and septum in mutants. Abbreviations: b: bladder; g: gonad and gonadal duct; hg: hindgut; k: kidney; u: urachus. The urogenital septum is indicated by an asterisk.

To study the visceral component in our mutants with *sirenomelia*, we first performed a gross anatomy dissection of the abdominal organs in newborn specimens (Fig. 3). The macroscopic dissection showed absence of obvious anomalies in the liver, stomach and midgut. We focused our analysis on the *Bmp7^−/−^;Shh^+/−^* genotype because the milder phenotype makes it more informative. In *Bmp7^−/−^;Shh^+/−^* newborns the gross anatomy malformations concentrated in the urogenital track and hindgut. The kidneys and ureters were always severely affected, highly hipoplastic and frequently hydronephrotic and the bladder was consistently absent (Fig. 3A–B). The hindgut was narrower than normal and frequently ended blind. In some cases the intestine and stomach were distended with gas (not shown) probably secondary to the anal atresia and/or to a possible tracheo-esophageal fistula as has been shown for *Bmp7* mutants [45]. In contrast, the gonads and adrenal glands were easily identifiable and no obvious gross abnormalities of the gonadal ducts of either sex were observed (Fig. 3A–B; scheme).

A careful histological analysis of serial sagittal sections at E12.5 and E15.5 (Fig. 3) confirmed the results of the anatomic dissection in newborns. Whereas the urogenital septum separated the urogenital and anorectal tracts in wild type embryos (asterisk in Fig. 3C), it was never observed in mutants. Only the atrophic and frequently ending blind hindgut was distinguishable in *Bmp7^−/−^;Shh^+/−^* mutants (Fig. 3D). Interestingly, a blind termination of the urachus (an epithelial duct connecting the allantois and the bladder), due to the absence of bladder was also observed (Fig. 3D).

Thus, the spectrum of visceral malformations exhibited by *Bmp7^−/−^;Shh^−/−^* and *Bmp7^−/−^;Shh^+/−^* mutants faithfully recapitulates that reported in human cases of *sirenomelia*. Finally, it should be pointed out that *Bmp7^−/−^;Shh^−/−^* and *Bmp7^−/−^;Shh^+/−^* mutants also display the traits typical of the *Bmp7* deficiency including microphtalmia, heart and thoracic skeletal defects (not shown) [42]. In this regard, human *sirenomelia* has been also associated with malformations of the cranial body [4].

### Molecular defect in the *sirenomelia* mutants

The phenotype of our mutants must result from reduced Bmp and Shh signaling. To understand and localize the origin of the defect, we first investigated the pattern of expression of *Shh* and *Bmp7* during the early normal embryonic development of the caudal body.

**Figure 4:**
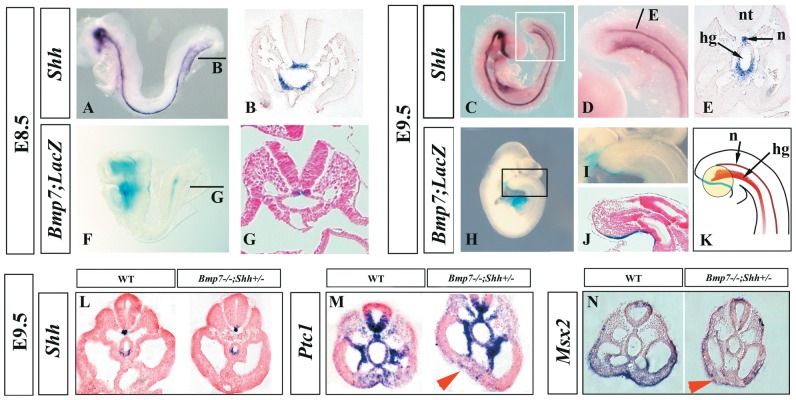
Reduced Bmp7 and Shh signaling in the ventral caudal mesoderm of mutant embryos. (A–E) Expression of *Shh* in whole mount ISH and corresponding transverse section (level indicated by bar) at E8.5 (A–B) and E9.5 (C–E). (F–J) Expression of *Bmp7* by Bmp7-LacZ reporter in *Bmp7* ^+/−^ embryos at E8.5 (F–G) and E9.5 (H–J). (K) Cartoon showing the pattern of expression of *Shh* and *Bmp7* at E9.5, the area influenced by both signals is encircled. (L–N) Expression of *Shh* (L), *Ptc1* (M) and *Msx2* (N) in transverse caudal sections of E9.5 wild type and *Bmp7^−/−^;Shh^+/−^* embryos as indicated at the top of each panel. Abbreviations: c: coelomic cavity; da: dorsal aorta; hg: hindgut; n: notochord; nt: neural tube; o: omphalomesenteric artery.

*Shh* expression was analyzed by whole mount in situ hybridization (ISH) followed by section analysis. As previously described, *Shh* transcription occurred in the notochord and ventral hindgut [38], both at E8.5 (10–12 pairs of somites; before the turning of the embryo) and at E9.5 (Fig. 4A–E).

As the *Bmp7* null allele carries LacZ [37], [41], we used X-gal staining in *Bmp7* heterozygous embryos to detect *Bmp7*-expressing cells. At E8.5, *Bmp7* expression was restricted to the notochord as can be observed in the whole mount staining and in corresponding transverse section (Fig. 4F–G). At E9.5, *Bmp7* expression was mainly detected in the ventral ectoderm, caudal to the allantois, and corresponding to the level of the cloaca as can be appreciated in the whole-mount staining and medial sagittal section (Fig. 4H–J) [46], [47]. This area of expression overlaps the ventral ectodermal ridge (VER), a remnant of the primitive streak [46]. A faint X-gal staining also remained in the notochord at this stage. A scheme depicting the pattern of expression of *Shh* (redish) and *Bmp7* (blue) at E9.5 is shown in Fig. 4K.

To identify the state of Bmp and Shh signaling in our mutants, we compared the expression of well-established downstream targets of each pathway in wild type and mutant embryos at E9.5 focusing on the caudal embryo body. First, as expected for *Shh* heterozygous, we found that the domain of *Shh* expression was similar in *Bmp7^−/−^;Shh^+/−^* mutants and wild type embryos (Fig. 4L). The expression of *Ptc1* was used as reporter of Shh activity. In wild-type embryos, *Ptc1* transcripts were detected in the ventral neural tube and notochord, medial somites, splanchopleure, hindgut and the ventral caudal mesoderm in control embryos (Fig. 4M). In *Bmp7^−/−^;Shh^+/−^* mutant embryos, *Ptc1* expression in the ventral caudal mesoderm was clearly downregulated suggesting a decrease in hh signaling at this level (Fig. 4M). The expression of *Msx2*, a bona fide target of Bmp signalling, that normally occurs in the ventral caudal mesoderm and ectoderm of control embryos, was dramatically downregulated in *Bmp7^−/−^;Shh^+/−^* embryos (Fig. 4N). The downregulation in *Msx2* in compound mutants reflects the loss of *Bmp7* signalling as is similarly observed in *Bmp7* single mutants but not observed in *Shh* single mutants (not shown). Also, the expression of *Bmp4* and *Bmp2* ligands was not significantly changed in our mutants (Fig. S2 and not shown).

Thus, our analysis localizes the region with defective signaling in the ventral caudal mesoderm between the allantois and the cloaca. This corresponds to the pericloacal ventral region and is encircled in Fig. 4K.

### Onset of the mutant phenotype

To get insights into the way the mermaid phenotype was established, we performed a histological analysis in our allelic series of embryos. The analysis focused on the caudal body, and was performed at E8.5 (10–12 Somites) and E9.5 (22–23 Somites) before posterior morphogenesis was heavily altered. For a maximum detail, it was performed in semithin (1 micron thick) transverse serial sections of araldite embedded embryos.

**Figure 5:**
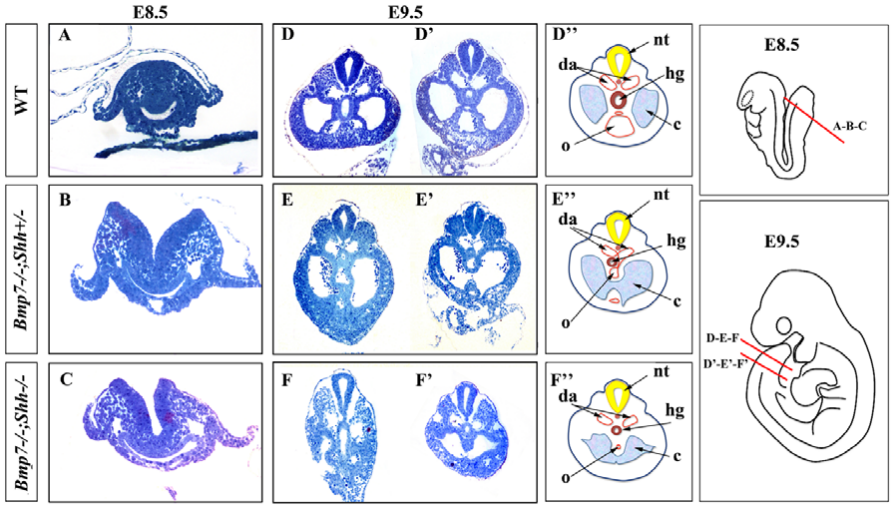
Onset of the morphology defect. Semithin (1 micron thick) transverse serial sections stained with toluidine blue of control and mutant embryos (genotype indicated on the left) at E8.5 (A–C) and E9.5 (D–F′). The level of the section is indicated in the pictures on the right. (A–C) Sections at the tip of the tailbud of E8.5 embryos. No differences between wild type and mutants are observed. (D–F′) Section at two different caudal levels, as indicated on the right. Note the narrower hindgut and the absence of the recurved portions of the dorsal aorta in mutants. (D″–F″) Schematic representation of the sections with the structures marked. Abbreviations: hg:hindgut; da: dorsal aortas; nt: neural tube; c: choelomic cavity; o: omphalomesenteric artery. The presence of red blood cells in the coelomic cavity of mutants is an artifact that sometimes occurs when dissecting the embryo disregarding the genotype.

Transverse sections through the caudal region of E8.5 *Bmp7^−/−^*; *Shh^+/−^* and *Bmp7^−/−^*; *Shh^−/−^* embryos failed to detect any abnormality compared with equivalent stage wild type embryos (Fig. 5A–C). At this stage, the hindgut, the dorsal aortas and the primitive omphalomesenteric artery, a single vessel that forms immediately ventral to the hindgut as the splanchnopleural folds coalescence and fuse, showed similar morphology in all genotypes.

However, morphological differences between wild type and mutant embryos were evident by E9.5 (Fig. 5D–F″). In wild type embryos, transverse sections at the most caudal level showed the centrally positioned cloaca, the bilateral coelomic cavities and the dorsal aortas and their ventral recurved portions (Fig. 5D). A section through a more cranial level revealed the omphalomesenteric artery derived from the fusion of the two ventral recurved aorta portions (Fig. 5D′). The omphalomesenteric artery that, at this stage, was of big caliber, contacted the hindgut dorsally and the ventral body wall ventrally (Fig. 5D′) dividing the coelomic cavity into two independent bilateral cavities, at this low abdominal (pelvic) level.

Our study revealed similar defects in *Bmp7^−/−^*;*Shh^−/−^* and *Bmp7^−/−^*;*Shh^+/−^* mutants at this stage. First, the diameter of the mutant hindgut and cloaca was significantly reduced compared to wild type embryos (Fig. 5E–F compare with Fig. 5D). In fact, the final dilatation typical of the cloaca was only observed in much fewer sections than in wild type littermates indicating its poor development (Fig. 5E–F). Second, the final recurved portions of the paired dorsal aortas were not identified in the mutants and neither was the omphalomesenteric artery derived from their fusion (Fig. 5E–E′, F–F′). In contrast, the omphalomesenteric artery continued ventral to the hindgut, as in previous stages. It showed a variably poor development and in the majority of cases did not contact the ventral body wall thus leaving the coelomic cavity undivided in mutants (Fig. 5E′–F′).

### Abnormal caudal vascular development in *sirenomelia* embryos

**Figure 6:**
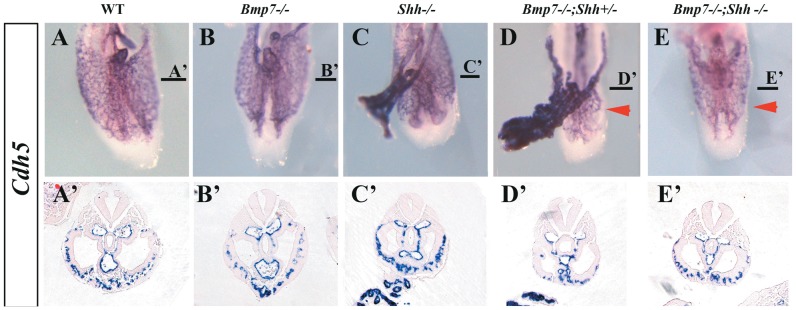
Expression of *Cadherin5* in wild-type and mutant embryos at E9.5. (A–E) Ventral views of the caudal region of E9.5 embryos hybridized for *Cadh5*. (A′–E′) Transverse sections of the hybridized embryos at the level indicated by the horizontal bar. The genotypes are indicted at the top. The red arrowheads point to the poorly local ventral mesoderm vascular plexus in *Bmp7^−/−^;Shh^+/−^* and *Bmp7^−/−^;Shh^−/−^* mutants.

The above-described vascular phenotype was confirmed by the expression of a specific endothelial marker, *cadherin5* (Cdh5, also VE-cadherin, [48]), in whole mount ISH followed by section analysis of hybridized embryos (Fig. 6). The expression of *Cdh5* marks the distribution of endothelial cells and revealed the poor development of the local plexus of the caudal ventral body wall in compound mutants (arrowheads in Fig. 6D–E). The recurved portions of the aorta were not identified and the omphalomesenteric artery was always narrower and dorsally positioned in the compound mutants than in the other genotypes (Fig. 6A′–E′). It should be noted that the morphology of the allantois was normal in the complete genotypic series.

In summary, our results demonstrate that *sirenomelia* has an early onset as the defects are present at E9.5. There is a concomitant impact on the hindgut, which is narrower than normal and shows a reduced cloacal dilatation, and in the caudal development of the dorsal aortas that fail to elongate and form the recurved portions. As a result the caudal midline of the embryo results highly affected.

### Analysis of cell death and cell proliferation

Changes in cell proliferation or abnormal cell death in mesodermal precursors could in principle explain the hindgut and vascular phenotype. Indeed, a shortage in mesenchymal cells is considered the primary gut defect in *Shh* mutants [49]. Therefore, we performed a sequential analysis of cell death and proliferation during the time the phenotype is being generated (E8.5 and E9.5). The TUNEL assays in transverse sections failed to detect differences in the amount or distribution of apoptotic cells between wild type and mutant embryos at E8.5 (Fig. S3). By E9.5, the analysis of serial transverse sections showed some scattered apoptotic cells in the hindgut and in the ventral caudal mesoderm of single *Bmp7^−/−^*, single *Shh^−/−^* and double *Bmp7^−/−^*; *Shh^+/−^* mutants while no cell death was detected in these locations in wild type embryos (Fig. S3). At this stage, cell death was more prominent in double homozygous *Bmp7^−/−^*;*Shh^−/−^* embryos all across the dorso-ventral midline of the embryo (Fig. S3).

To study cell proliferation we processed sections for anti-phosphorylated histone H3 immunohistochemistry that detects cells in mitosis. Our analysis failed to detect obvious differences between wild type, single and double mutants at E8.5 (not shown) and E9.5 (Fig. S4).

The finding that *Bmp7^−/−^*; *Shh^+/−^* mutants, which display *sirenomelia*, only showed a minimal increase in cell death intensity suggests that abnormal cell death or proliferation are not a major cause for the phenotype. Nevertheless, differences in proliferation or cell death of a few critical precursors at an undefined early stage may still occur.

### Normal development of the leg buds except for their merging

It has already been suggested that the fusion of the lower legs in *sirenomelia*, although giving the name to the condition, is secondary to the absence of the midline structures that usually separate the left and right hindlimb fields [16], [50], [51]. Our results support this hypothesis.

**Figure 7:**
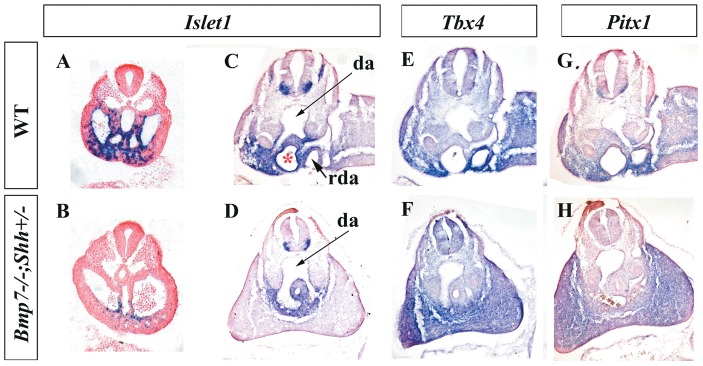
Spatial distribution of *Islet1*, *Tbx4* and *Pitx1* mRNA in wild-type and mutants. (A–D) *Islet1* expression in transverse sections through the caudal region of E9.5 (A–B) and 10.5 (C–D) wild type and mutant embryos. Note the strong downregulation of expression in the midline tissues and in the interlimb mesoderm of mutants. Note the absence of the recurved portions of the dorsal aortas in E10.5 mutant embryos. (E–H) Expression of *Tbx4* (E–F) and *Pitx1* (G–H) specific hindlimb markers in consecutive sections of E10.5 wild-type and mutant embryos showing the abnormal connection between both hindlimbs. Note the absence of the recurved portions of the aorta in mutants. Genotypes indicated on the left. Da: dorsal aorta; rda: recurved portion of aorta.

To explore the situation of the interlimb region, we analyzed the expression of *Islet1* (*Isl1*), a gene that is expressed in this location and that is important for hindlimb development. Isl1 is a LIM homeodomain transcription factor with multiple functions in cardiac, neural and hindlimb development [52], [53], [54], [55], [56]. It has been shown that its expression is regulated by Shh in neural tissues [57] and by Bmp4 in the oral epithelium [58]. Interestingly, *Isl1* expression was highly downregulated in the caudal body of *Bmp7^−/−^*;*Shh^+/−^* mutants compared with wild-type littermates (Fig. 7A–B). At E9.5 *Isl1* transcripts were found in the ventral hindgut, splanchopleure and ventral interlimb mesoderm of wild-type embryos whereas only some transcripts were detected in the splanchopleure of mutants. At E10.5 *Isl1* was prominently expressed in the interlimb tissue particularly around the cloaca and recurved portions of the aortas (Fig. 7C) whereas in mutant embryos the expression was much reduced around the hindgut and in the splanchopleure (Fig. 7D). These results support the hypothesis that the midline tissues including the interlimb ventral body wall are not present in *sirenomelia* (Fig. 7D, compare with Fig. 7C).

We next examined *Tbx4* and *Pitx1* expression, specific markers of the hindlimb mesenchyme [54]. At E10.5 both *Tbx4* and *Pitx1* were conspicuously expressed in the hindlimb mesoderm and interlimb anterior mesoderm of wild-type embryos (Fig. 7E, G). In *sirenomelia* mutants, the expression of *Tbx4* and *Pitx1* occurred in a continuous domain reflecting the joining of both hindlimbs (Fig. 7F, H).

**Figure 8:**
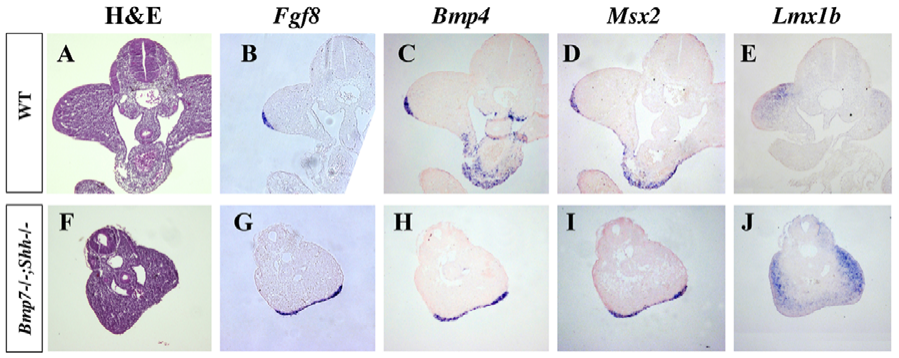
Gene expression analysis in hindlimbs of wild-type and *Bmp7^−/−^;Shh^−/−^* littermates at E10.5. The expression of limb bud markers is normal in mutant hindlimbs except for the merged phenotype. The domain of expression of *Fgf8* (G), *Bmp4* (H) and *Msx2* (I) in the apical ectodermal ridge of both hindlimb is continuous because of the merging. The expression of *Lmx1b* reflects the abnormal position of the limbs (J–E).

Except for their fusion, the development of the hindlimbs followed a normal course. The expression of *Fgf8*, a marker of the apical ectodermal ridge (AER) that is a signaling center crucial for limb development, was detected in mutants in a continuous stripe (shown for homozygous mutants in Fig. 8G, compare with wild-type in Fig. 8B) as the fusion of the leg buds along their posterior border resulted in the continuation of the AERs of both hindlimbs. *Bmp4* and *Msx2* were normally expressed both in the AER (Fig. 8 C–D and Fig. 8H–I) and mesoderm (not shown) of double homozygous mutants. Finally, *Lmx1b* expression, a marker of the dorsal mesoderm, also revealed the abnormal dorso-ventral position of the fused hindlimbs (Fig. 8E and J). These results allow the conclusion that limb development is not impaired in *sirenomelia* in agreement with the complete hindlimb development exhibited by *Bmp7^−/−^*;*Shh^+/−^* newborns (Fig. 1D–F and Fig. 2. C). The impaired final limb phenotype of double homozygous (*Bmp7^−/−^*;*Shh^−/−^*) corresponds to that of the *Shh* deficit with the above described improvement at zeugopod level due to the decreased in Bmp signaling [43], [44].

## Discussion

### A new animal model for human *sirenomelia*

Our results show a genetic interaction between *Bmp7* and *Shh* that results in a spectrum of phenotypes including leg fusion and visceral malformations that are morphologically identical to human cases of *sirenomelia*. Based on the similarity of the phenotypes we consider that *Bmp7^−/−^*;*Shh^−/−^* and *Bmp7^−/−^*;*Shh^+/−^* mutants conjointly constitute an excellent animal model in which study the mechanisms involved in the development of this devastating human malformation. Because double homozygous associates the traits typical of the *Shh* deficiency (Cyclopia, proboscide, abscence of ribs… [38]), they may be a less suitable model although it is important to note that human cases of *sirenomelia* with cyclopia have been reported, including a recent cluster in Cali Colombia [9], [28], [59], [60].

This novel animal model has allowed us to investigate the onset of the *sirenomelia* malformation. Our results show a defective formation of midline structures in the caudal embryonic region coincident with the area in which decreased Bmp and Shh signaling is detected. The signaling defects result in a hypoplastic hindgut and precludes the appropriate vasculogenesis of the caudal end of the dorsal aortas and the remodeling of the primitive vascular plexus. The atrophic hindgut and cloaca, together with the vascular defect, results in a defective midline that leads to the junction of the limb fields.

### The merging of the lower limbs is a secondary event in *sirenomelia*

Our results strongly suggest that the approximation and merging of the hindlimb fields are the result of the early visceral defect that leads to a considerably midline reduction. Therefore, the limb fusion phenotype, although giving the name to the condition, is a secondary event in *sirenomelia* as it has already been proposed [16], [50], [51].

The diagnosis of *sirenomelia* is certain since it relies on the fusion of the lower limbs. The grade of lower limb fusion considerably varies generally in correlation with the severity of the visceral associated malformations [3], [10]. The mildest cases, types I and II of Stocker and Heifetz classification, are characterized by the midline merging of two almost normal legs except for their malposition. The most severe cases, types VI and VII of Stocker and Heifetz classification, are characterized by a single central tapering appendage that misses clear leg features. These two extreme phenotypes are seen in our mutants: *sirenomelia* type I and II is typically found in *Bmp7^−/−^;Shh^+/−^* mutants, while type V is typically found in *Bmp7^−/−^;Shh^−/−^* double homozygous. Interestingly, the mild phenotypes show that the approximation and merging of the lower limbs do not interfere with normal limb morphogenesis except for the absence of rotation. This conclusion is supported by the gene expression study of limb development we have performed in the double mutants. Our analysis also indicates that the most severe lower limb phenotypes may result from late deficits in limb development independent of the merging event. For example, in our mutants, the deficit in Bmp and Shh signaling has an early impact in generating *sirenomelia* and an additional later effect in limb development.

One particularly interesting feature of the phenotype, which is constantly observed in all cases of *sirenomelia*, is the abnormal position of the legs with the soles of the feet facing anteriorly and the great toes in a lateral position. This position corresponds to a 180° rotation with regard to the normal position and results from the absence of the rotation that hindlimbs normally experience during embryonic development. Very little is known about the mechanisms that direct this rotation, but it seems obvious that the merging of the limb buds would prevent them from operating. This lack of rotation may also contribute to the altered pelvic morphology of our mutants, with both ischia fused in the midline and the pubes laterally separated. In wild type embryos, the two bilateral hip bones progressively join in the anterior midline forming the pubic symphysis by E16.5 [61], [62], [63]. In our mutants, the approximation of both pubes to the midline does not occur and therefore the pubic symphysis does not form whereas both ischia fuse in the midline. Thus, it seems reasonable to propose that the early merging of the leg buds would preclude the normal morphogenetic movements that reposition the pelvic girdle and lower limb and therefore leading to the observed phenotype.

### A reevaluation of the etiopathogenic hypotheses of *sirenomelia*

As a result of the clinical studies of series of fetuses and newborns with *sirenomelia*, two main hypotheses have been proposed to explain this devastating malformation. First, based on the general impact in the caudal body, *sirenomelia* is envisaged as resulting from a deficient blastogenesis that failed to produce sufficient mesodermal precursors [3], [8], [14]. Second, based on the almost constant abnormal development of the umbilical vessels, *sirenomelia* is considered the result of a vascular defect that leaves the lower part of the body with a severely deficient circulation incompatible with normal development [2], [21], [64], [65].

According to the deficient blastogenesis hypothesis, *sirenomelia* may be considered as a severe form of caudal dysgenesis (CD). CD is a heterogeneous assemblage of caudal anomalies that always include some degree of sacral agenesis [17], [20]. The milder *sirenomelia* phenotype of *Bmp7^−/−^;Shh^+/−^* mutants, although associating major visceral defects in the urogenital and low intestinal tracts, exhibits a normal development of the sacrum and tail. This allows the conclusion that *sirenomelia* may exist as a pathologic entity distinct from CD. Interestingly, human cases with minimum or even absent dorsal skeletal and neurological component have also been reported [66], [67].

Sirens resulting from reduced Bmp signaling (*Bmp7;Tsg* and our mutant) exhibit minor, if any, malformation of the sacrum and tail indicating normal anterior-posterior axis elongation. However, sirens resulting from excess RA signaling present impaired elongation of the caudal body [25], [68] and therefore they could be considered part of the CD. These observations indicate the existence of at least two different presentations of *sirenomelia* depending on the presence of dorsal malformations and suggest that different regions (dorsal and ventral) of the caudal body may be subject to different developmental regulation. Some of these signals may be restricted to particular caudal regions while others may have a broader effect. Bmp and Shh signaling appear to be particularly important for the development of the ventral midline and body wall while Wnt signaling and RA signaling may have a more general effect. Thus, the heterogeneity in *sirenomelia* clinical presentations likely reflects different etiologies that distinctly affect different signaling pathways.

Regarding vascular steal hypothesis, our study reveals an early vascular defect in *sirenomelia*. We have detected a failure in the formation of the recurved portions of the aortas that leads to a misorganization of the ventral caudal vasculature. This abnormal arrangement of the caudal vessels is likely the origin of the abnormal Single Umbilical Artery (SUA) described in human cases of *Sirenomelia* [21], [65], [69]. This results from the persistence of the primitive omphalomesenteric artery due to the failure or underdevelopment of the recurved portions of the aorta. However, our data do not support a vascular origin for *sirenomelia* since the concomitant onset of the vascular and visceral defects precludes a causal relationship. The absence of the recurved portions of the aorta certainly contributes to the reduction of midline structures and subsequent merging of the hindlimb fields but no through a hypoperfusion mechanism as originally proposed by the vascular steal hypothesis.

To sum up, our study and the available experimental and human data fit with a genetic origin of *sirenomelia* that affects some of the signaling pathways involved in the development and organization of the pelvic organs and ventral body wall. The fact that each of these pathways involves multiple components, it provides an explanation for the genetic heterogeneity and phenotypic variability.

### Bmp signaling and *sirenomelia*

*Sirenomelia* was first associated with reduced Bmp signaling when it was reported that the compound *Bmp7;Tsg* mutants exhibited this phenotype [26]. It should be noted that single *Bmp7* mutants do not exhibit a mermaid phenotype and that a further reduction in Bmp signaling caused by the removal of one or two functional copies of the Bmp positive modulator *Tsg* is required to get the phenotype [26], [41], [70]. Also, it has been shown that the removal of the *BMP receptor 1a* (*Bmpr1a*), specifically from the interlimb mesoderm with the *Isl1Cre* line, leads to the approximation of both hindlimbs, but it does not to *sirenomelia* [53]. Thus, an appropriate level of Bmp signaling seems to be absolutely required for normal ventral caudal mesoderm development. This requirement is conserved across species as it has been confirmed in Xenopus laevis and in zebrafish [26], [71].

The similarity of the *sirenomelia* phenotype of our mutants and that of *Bmp7;Tsg* mutants suggest a common origin. Bmps are well-accepted targets of Shh in several systems including the gut [72], [73], [74], [75]. Therefore, it is possible that the decrease of *Shh* dose in the absence of *Bmp7* results in a further reduction of Bmp signaling similarly to the double *Bmp7;Tsg* mutant. However, our gene expression analysis do not support this hypothesis because the expression of *Msx2* is similarly reduced in *Bmp7* single and *Bmp7*;*Shh* compound mutants. Furthermore, a reduction in Shh signaling could contribute by itself to hindgut and vascular malformations [49], [76], [77]. Therefore, we favor the interpretation that the *sirenomelia* phenotype in our mutants is the result of the summation effect of reduced Bmp and reduced Shh signaling. Interestingly, the double *Tsg^−/−^;Bmp4^+/−^* mutant presents holoprosencephaly, a characteristic of *Shh* loss of function [38], [78]. Accordingly, Shh expression was undetectable in the fore and midbrain of *Tsg^−/−^;Bmp4^+/−^* mutants, explaining the holoprosencephaly phenotype. Thus, the possibility that *Shh* function is reduced in *Bmp7;Tsg sirenomelia* mutants should be explored. In any case, the crosstalk between Bmp and Shh signaling pathways in the development of the caudal body merits further investigation.

Besides, the co-localization of the anatomic vascular defect and the site of maximum signalling defect suggest a causal relationship. Particularly, the progression of the dorsal aorta is thought to occur by the direct assembly of endothelial precursor cells (vasculogenesis) under the influence of paracrine signals that regulate the positioning and remodeling of embryonic blood vessels. Interestingly, members of the hedgehog family [77], [79], [80] and bone morphogenetic proteins (BMP) [81], [82] are among the best-known paracrine factors that promote vascular development.

Collectively, our findings provide compelling evidence that the vasculogenesis of the recurved portions of the dorsal aortas and the reorganization of the vessels in the pericloacal area and ventral mesoderm caudal to the umbilicus require appropriate Bmp and Shh signaling. This is a region in which appropriate signaling is absolutely required to permit the normal development and organization of pelvic organs and ventral body wall.

### Implications for human *sirenomelia*

Our data support the notion that the primary morphological defect in *sirenomelia* is an insufficient formation of midline structures, mainly the hindgut and caudal vasculature. This fits very well with the phenotypes observed in human cases of *sirenomelia* and contributes to a better understanding of the mechanisms that lead to this malformation.

Although most human cases of *sirenomelia* are sporadic, the information lately gathered from animal models, including our model, suggests a complex multigenetic basis for *sirenomelia*, probably requiring several genetic defects even in heterozygosis. This can explain the variability in phenotypic traits as well as the low prevalence in human populations. The recent report of familial descriptions of *sirenomelia* further supports this notion (27).

Our study also helps managing the malformations of the caudal body by determining candidate genes to establish screenings or develop markers to assist identifying potential couples at a higher risk of having an infant with caudal defects. We anticipate that in the future it will be particularly important to assess the contribution of human *BMP7* gene to the genetic risk for that spectrum of developmental defects.

## Supporting Information

Figure S1 **Comparison of zeugopod development in** ***Shh^−/−^*** **versus double** ***Bmp7^−/−^;Shh^−/−^*** **mutants.** Alizarin Red-Alcian Blue skeletal preparations are accompanied by an schematic representation of the skeletal elements. Note the improvement in the morphology of the zeugopod in the double mutant compared to that of the single *Shh^−/−^* mutant. Abbreviations: f:fibula; t:tibia; Z: zeugopod.
(TIF)

Figure S2 **Expression of** ***Bmp4*** **in E9.5 embryos of the principal genotypes of the** ***Bmp7;Shh*** **allelic series.** Note similar *Bmp4* expression in the caudal ventral mesoderm disregarding the genotype. Genotypes indicated at the top.
(TIF)

Figure S3 **Cell death analysis.** TUNEL assay in transverse sections of E8.5 and E9.5 wild type and mutant embryos as indicated at the top of each panel.
(TIF)

Figure S4 **Cell proliferation analysis.** The immunohistochemistry with anti pH3, which marks cells in mitosis, failed to detect obvious differences between genotypes.
(TIF)

Table S1 **Characterization of external phenotype of neonates obtained from** ***Bmp7^+/−^;Shh^+/−^*** **intercrosses.** Table showing the number and percentage of external malformations observed in neonates of the allelic series. (*) Cyclopia and regressed tail due to Shh-null background.
(DOCX)
