# Supplementary figures and images for: Sirenomelia Phenotype in Bmp7;Shh Compound Mutants: A Novel Experimental Model for Studies of Caudal Body Malformations
Source: PLoS One. 2012 Sep 17;7(9):e44962. doi: 10.1371/journal.pone.0044962 (PMC3444499; doi:10.1371/journal.pone.0044962)

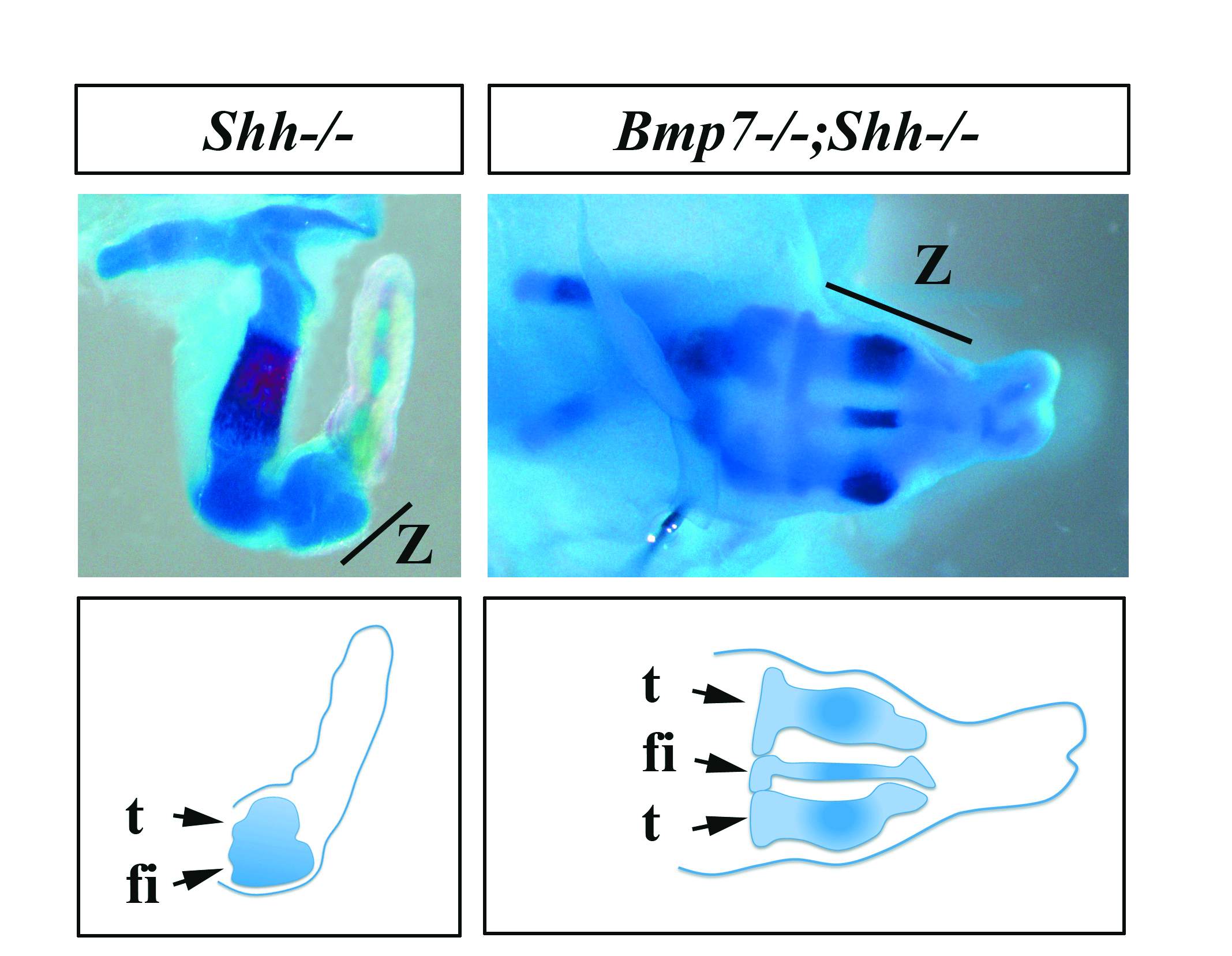

Supplement: Figure S1 — Comparison of zeugopod development in Shh−/− versus double Bmp7−/−;Shh−/− mutants. Alizarin Red-Alcian Blue skeletal preparations are accompanied by an schematic representation of the skeletal elements. Note the improvement in the morphology of the zeugopod in the double mutant compared to that of the single Shh−/− mutant. Abbreviations: f:fibula; t:tibia; Z: zeugopod. (TIF) [file pone.0044962.s001.tif]

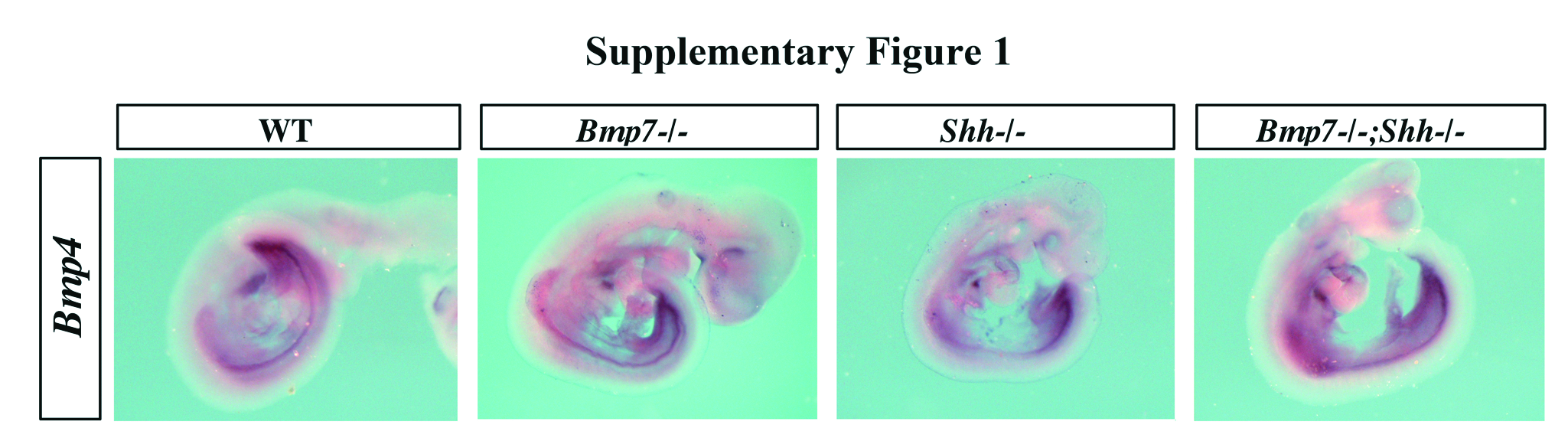

Supplement: Figure S2 — Expression of Bmp4 in E9.5 embryos of the principal genotypes of the Bmp7;Shh allelic series. Note similar Bmp4 expression in the caudal ventral mesoderm disregarding the genotype. Genotypes indicated at the top. (TIF) [file pone.0044962.s002.tif]

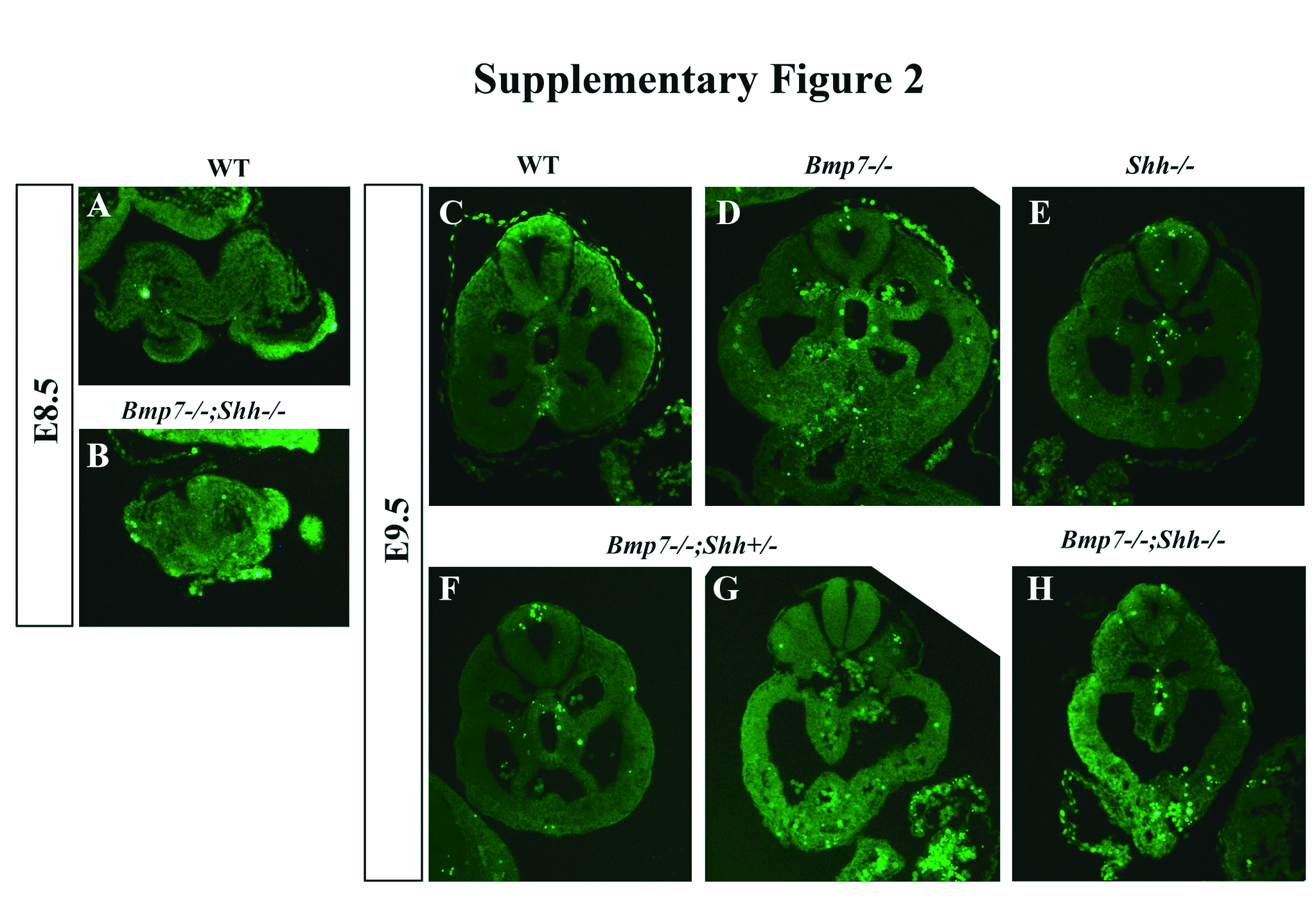

Supplement: Figure S3 — Cell death analysis. TUNEL assay in transverse sections of E8.5 and E9.5 wild type and mutant embryos as indicated at the top of each panel. (TIF) [file pone.0044962.s003.tif]

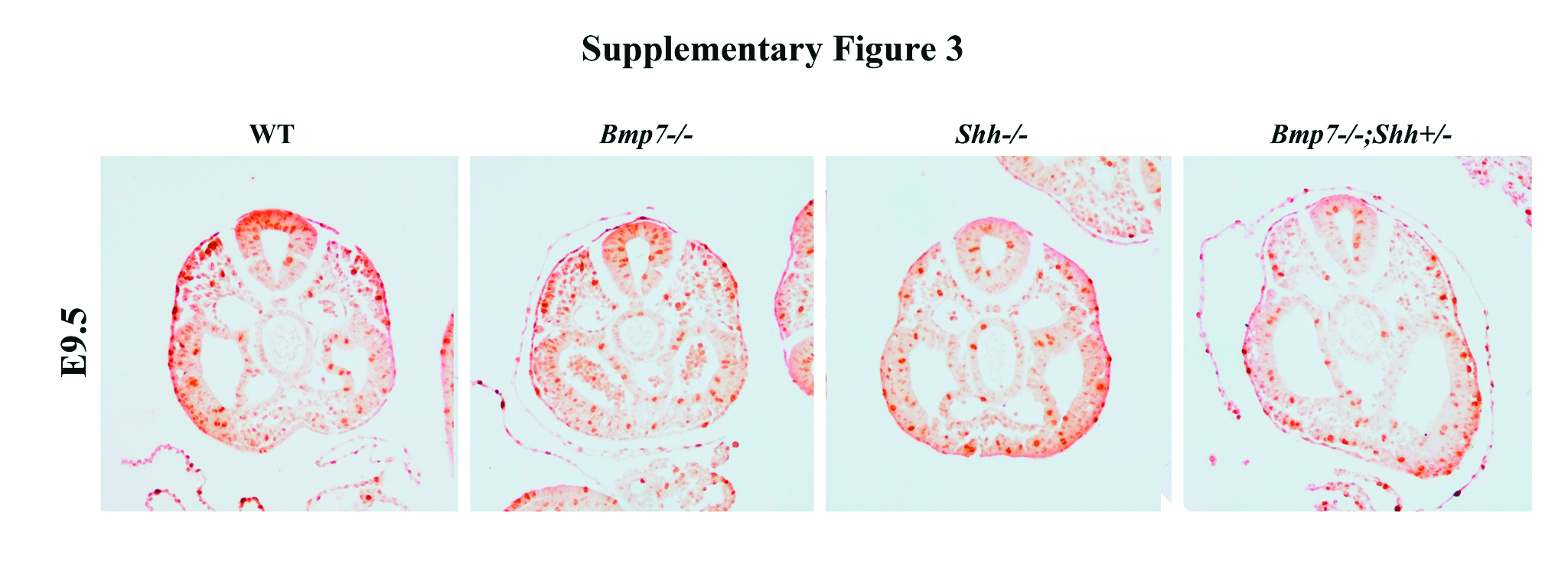

Supplement: Figure S4 — Cell proliferation analysis. The immunohistochemistry with anti pH3, which marks cells in mitosis, failed to detect obvious differences between genotypes. (TIF) [file pone.0044962.s004.tif]
