# Supplementary material for: Sirenomelia Phenotype in Bmp7;Shh Compound Mutants: A Novel Experimental Model for Studies of Caudal Body Malformations
Source: PLoS One. 2012 Sep 17;7(9):e44962. doi: 10.1371/journal.pone.0044962 (PMC3444499; doi:10.1371/journal.pone.0044962)
Supplement: Table S1 — Characterization of external phenotype of neonates obtained from Bmp7+/−;Shh+/− intercrosses. Table showing the number and percentage of external malformations observed in neonates of the allelic series. (*) Cyclopia and regressed tail due to Shh-null background. (DOCX) [file pone.0044962.s005.docx]

| Genotype | Neonates | Expected % | Observed % | Hindlimb Fusion (%) | Anal atresia (%) | Genital tubercule (%) | Microphtalmia (%) | Kinked Tail (%) |
| --- | --- | --- | --- | --- | --- | --- | --- | --- |
| Bmp7*^+/+^*Shh*^+/+^* | 18 | 6,25 | 10,58 | 0 (0) | 0 (0) | 18 (100) | 0 (0) | 0 (0) |
| Bmp7*^+/+^*Shh*^+/-^* | 25 | 12,5 | 14,71 | 0 (0) | 0 (0) | 25 (100) | 0 (0) | 0 (0) |
| Bmp7*^+/+^*Shh*^-/-^* | 1 | 6,25 | 0,59 | 0 (0) | 0 (0) | 0 (0) | 0* (0) | 0* (0) |
| Bmp7*^+/-^*Shh*^+/+^* | 29 | 12,5 | 17,05 | 0 (0) | 0 (0) | 29 (100) | 0 (0) | 0 (0) |
| Bmp7*^+/-^*Shh*^+/-^* | 52 | 25 | 30,58 | 0 (0) | 0 (0) | 52 (100) | 0 (0) | 0 (0) |
| Bmp7*^+/-^*Shh*^-/-^* | 11 | 12,5 | 6,47 | 0 (0) | 0 (0) | 0 (0) | 0* (0) | 0* (0) |
| Bmp7*^-/-^*Shh*^+/+^* | 10 | 6,25 | 5,88 | 0 (0) | 0 (0) | 10 (100) | 10 (100) | 8 (80) |
| Bmp7*^-/-^*Shh*^+/-^* | 21 | 12,5 | 12,36 | 11 (52) | 11 (52) | 10 (47) | 21 (100) | 18 (85) |
| Bmp7*^-/-^*Shh*^-/-^* | 3 | 6,25 | 1,76 | 3 (100) | 3 (100) | 0 (0) | 0* (0) | 0* (0) |
| Total | 170 |  | | | | | | |

**Table 1. Characterization of external phenotype of Neonates obtained from Bmp7+/-;Shh+/- intercrosses.**

* Cyclopia and regressed tail due to *Shh* null background [[36](#_ENREF_36)].
